# Sex ratio among offspring of childhood cancer survivors treated with radiotherapy

**DOI:** 10.1038/sj.bjc.6600748

**Published:** 2003-02-10

**Authors:** J F Winther, J D Boice, B L Thomsen, W J Schull, M Stovall, J H Olsen

**Affiliations:** 1 Institute of Cancer Epidemiology, Danish Cancer Society, Strandboulevarden, 49, DK-2100 Copenhagen, Denmark; 2 International Epidemiology Institute, 1455 Research Blvd, Suite 550, Rockville, MD 20850-6115, USA; 3 Vanderbilt University, Department of Medicine, Vanderbilt Medical Center and Vanderbilt-Ingram Comprehensive Cancer Center, Nashville, Tennessee, TN 37232, USA; 4 Human Genetics Center, School of Public Health, The University of Texas Health Science Center, 1200 Hermann Pressler Street, PO Box 20186, Houston, TX 77225, USA; 5 Department of Radiation Physics, The University of Texas, MD Anderson Cancer Center, Box 544, 1515 Holcombe Blvd., Houston, TX 77030, USA

**Keywords:** childhood cancer, sex ratio in offspring, population-based cohort study, epidemiology

## Abstract

It has been postulated that paternal gonadal exposure would increase the sex ratio by inducing X-chromosomal dominant lethals but that maternal gonadal exposure would decrease the sex ratio by inducing recessive sex-linked lethals. We therefore evaluated the sex ratio (male-to-female ratio) of children born to survivors of childhood cancers in Denmark. Children with cancer were identified from the Danish Cancer Registry from 1943 to 1996 and their offspring from the Central Population Registry. Radiation treatments were determined from records within the Cancer Registry and gonadal radiation exposures were estimated based on the cancer being treated and the likely proximity of the radiation fields to the gonads. Overall, 1100 survivors of childhood cancer became the parents of 2130 children. The sex ratio for male (0.99) and female (1.00) cancer survivors was similar and did not differ significantly from the Danish population (1.06). Radiotherapy did not influence the sex ratio of the children of either male or female survivors, and there was no evidence for dose-related changes over categories of estimated dose to parental gonads. We saw no consistent association between the sex ratio and the interval between cancer diagnosis of the parent and birth of the child. This nationwide study provides no support for the hypothesis that radiation exposure to the gonads results in an inherited genetic effect that would be manifested by a change in the sex ratio of children born after exposure. It may be, however, that sex ratio alterations are not a good or even a valid indicator of possible genetic effects in humans.

In 1958, a possible alteration in the ratio of male-to-female births (the sex ratio) was suggested following parental exposure among survivors of the atomic bombings of Hiroshima and Nagasaki (Schull and Neel, 1958). The changes in the sex ratio were in the direction expected if exposure had resulted in the induction of sex-linked lethal mutations (Neel, 1963). However, an extended study published in 1966 that increased the sample size by about 70% to 47624 children of parents exposed to atomic radiation failed to confirm the earlier finding of a change in the sex ratio of the offspring of the survivors (Schull *et al*, 1966). Recognition of the sex chromosome aneuploidy and the Lyonisation effect has brought into question the utility of the sex ratio as an indicator of an inherited radiation-induced genetic effect (Neel *et al*, 1990; Neel and Schull, 1991). Nonetheless, there continues to be an interest in evaluating sex ratio following paternal preconceptional radiation with inconsistent results–the most recent studies evaluated more than 16 000 offspring of men employed at the Sellafield nuclear installation in Cumbria (Dickinson *et al*, 1996) and over 46 000 children born to nuclear industry workers in the UK (Maconochie *et al*, 2001).

Studies of the offspring of survivors of childhood and adolescent cancer offer a unique opportunity to evaluate whether preconception radiation can alter the sex ratio. Gonadal doses are of sufficient levels (typical between 0.01 and 2 Gy from scatter radiation) (Boice *et al*, 2002, unpublished data) and sample sizes are sufficiently large to have adequate power (Boice *et al*, 2000). Furthermore, the results of such high dose studies can be contrasted with the lower dose studies of occupationally or environmentally exposed populations (Dickinson *et al*, 1996; Maconochie *et al*, 2001; UNSCEAR, 1993,^2001^).

Previous studies on the sex ratio among offspring of survivors of childhood cancer have provided little evidence for an alteration following curative therapies (Hawkins, 1991; Byrne *et al*, 1998). In a large and comprehensive nationwide population-based cohort study using the unique population and health registries within Denmark, we have investigated whether radiotherapy received by childhood cancer patients affected the sex ratio of their offspring.

## MATERIAL AND METHODS

### Identification of childhood cancer survivors

Files of the national Danish Cancer Registry were used to identify all patients diagnosed with cancer before 20 years of age from 1943 to 1996. Included for study were those patients alive on or born after 1 April 1968, when the national Central Population Register (CPR) was established (and when all citizens were assigned a personal identification number). Throughout this paper, we will refer to these patients as childhood cancer survivors. The personal identification number, which is unique to every Danish citizen, incorporates date of birth and sex, and permits accurate linkage of information between registers. The Cancer Registry provided information on date of diagnosis, type of malignant neoplasm and information on radiotherapy on an yes/no level. Details on the registration and coding practices have been described previously (Danish National Board of Health, 1999). The 1100 cancer survivors (index patients) who met the inclusion criteria were grouped in one of the 12 main diagnostic tumour groups according to the international classification scheme for childhood cancer (Birch and Marsden, 1987).

### Identification of offspring

Offspring of index patients were identified through the CPR, which maintains a link between the personal identification numbers of parents and children. A total of 2130 children born in Denmark between 1 April 1968 and 1 January 2000 were included in the evaluation of the sex ratio.

### Radiation exposure

Information on radiotherapy (on an yes/no level) was available from notification forms in the Danish Cancer Registry. Notifications are forwarded to the Danish Cancer Registry from hospital departments when a case of cancer is diagnosed and when changes in the initial diagnosis and treatment (within 4 first months after diagnosis) occur (Danish National Board of Health, 1999). The likelihood of scattered radiation to the gonads was estimated based on the type of neoplasm being treated, knowledge of treatment procedures for specific tumours during the study period and assumptions of the gonadal proximity to the radiation field. Five categories of estimated radiation dose to the gonads were developed: *low, low or medium, medium, medium or high and high dose*. For some tumours, such as brain tumours, the scatter dose to gonads is very low whereas for other tumours, such as of gonadal origin, the radiation dose is high. The categories ‘low or medium’ and ‘medium or high’ were developed to mean that the true gonadal exposure could be in either of these two categories but sufficient details were not available to make the correct assignment.

### Statistical methods

Results are presented in terms of the offspring sex ratio – the ratio of the number of male births to the number of female births (M/F ratio) among survivors. To adjust for factors other than radiotherapy that might influence the sex ratio among offspring of childhood cancer survivors, the M/F ratio of non-irradiated parents was used as the referent category.

We conducted a log-linear analysis of the M/F ratio; that is, a logistic regression analysis with male sex of the child as the outcome variable. Separate analyses were performed for the offspring of male and female cancer survivors. The effect of radiation exposure was evaluated considering different proxy measures of exposure. The main variable was the information on radiotherapy (yes/no) obtained from the Danish Cancer Registry computerised records. Radiotherapy was considered as a single variable as well as in interaction models with other variables one at a time; that is, the 12 main diagnostic tumour groups, five categories of estimated gonadal radiation dose as described above, and the interval between cancer diagnosis of the parent and the birth of the offspring.

In the analyses, the following potential confounders were considered: age of mother, age of father and birth order of the child. Since adjustment for these variables did not affect the results, the unadjusted results are presented. Statistical tests are based on the likelihood ratio test statistic and confidence limits are based on Wald's test statistic on the log scale.

The trend test for categories of estimated gonadal radiation dose was performed in a model assuming a linear effect of the group number 1 (low) to 5 (high) on the log M/F ratio. The test of interaction between radiotherapy and interval between cancer diagnosis of the parent and birth of the offspring was performed modelling the effect of the exact interval on the M/F ratio as linear on the log scale, and testing whether significantly different trends occurred among the offspring of irradiated and non-irradiated parents. The number of male and female births in the general Danish population was available from published statistics (Statistics Denmark, 1997).

## RESULTS

The sex ratio among 2130 offspring of childhood cancer survivors (1056 offspring of 550 male survivors and 1074 offspring of 550 female survivors) is presented in Table 1

**Table 1:** M/F ratios among 2130 offspring^a^ of 1100 childhood cancer survivors, by sex of the survivor and exposure to radiotherapy^b^ compared to the general Danish population

**Study populations treatment**	**No. of offspring**	**No. of male offspring**	**M/F offspring ratio (95% CI)**	**M/F offspring ratio among irradiated parents divided by M/F offspring ratio among non-irradiated parents (95% CI)**
*Danish population*^c^	1 986 351	1 020 708	1.06	
*Childhood cancer survivors*				
Males (*n*=550)	1056	525	0.99 (0.88–1.16)	
Non-irradiated	665	334	1.01 (0.87–1.17)	
Irradiated	391	191	0.96 (0.78–1.16)	0.95 (0.74–1.22)
				
Females (*n*=550)	1074	538	1.00 (0.89–1.13)	
Non-irradiated	720	355	0.97 (0.84–1.13)	
Irradiated	354	183	1.07 (0.87–1.32)	1.10 (0.85–1.42)

M/F=male-to-female ratio. CI=confidence interval.

a Children born between 1 April 1968 and 1 January 2000.

b Information from the Danish Cancer Registry.

c Children born between 1968 and 1998.

. The sex ratios of the children born to both male (M/F ratio=0.99; 95% CI: 0.88–1.16) and female (M/F ratio=1.00; 95% CI: 0.89–1.13) survivors were slightly lower than the M/F ratio of 1.06 in the general Danish population. Radiotherapy did not affect the M/F ratio of the children of either male or female cancer survivors.

Table 2

**Table 2:** M/F ratios among offspring of childhood cancer survivors, by sex of survivor, main diagnostic tumour group (listed according to increasing estimated radiation dose to the gonads) and exposure to radiotherapy

				**M/F offspring ratio among**		
		**No. of offspring**		**M/F offspring ratios**		**irradiated parents divided by M/F offspring ratio**
**Main diagnostic tumour group (group no.)^a^**	**Sex of survivor (no.)**	**Total**	**Irradiated parents**	**Irradiated parents**	**Non-irradiated parents**	**among non-irradiated parents (95% CI)**
Central nervous system neoplasms (III)	M (102)	215	48	0.85	0.86	0.99	(0.52–1.88)
	F (122)	243	60	1.22	0.95	1.29	(0.72–2.32)
							
Sympathetic nervous system tumours (IV)	M (9)	16	4	1.00	1.40	0.71	(0.07–6.92)
	F (14)	24	18	1.00	2.00	0.50	(0.07–3.45)
							
Retinoblastoma (V)	M (26)	41	6	0.50	1.06	0.47	(0.08–2.92)
	F (37)	80	14	0.56	1.13	0.49	(0.15–1.63)
							
Malignant bone tumours (VIII)	M (27)	68	25	0.56	0.72	0.78	(0.28–2.16)
	F (22)	39	9	1.25	1.00	1.25	(0.28–5.59)
							
Hepatic tumours (VII)	M (1)	3	0	—	2.00	—	(—)
	F (1)	1	0	—	0.00	—	(—)
							
Leukaemias (I)	M (31)	53	9	2.00	1.32	1.52	(0.34–6.87)
	F (45)	78	26	1.36	0.93	1.47	(0.57–3.81)
							
Carcinomas and other malignant epithelial neoplasms (XI)	M (66)	125	25	1.50	1.22	1.22	(0.50–2.99)
	F (135)	292	55	1.04	0.94	1.10	(0.61–1.98)
							
Soft-tissue sarcomas (IX)	M (57)	118	28	0.75	1.00	0.75	(0.32–1.76)
	F (46)	85	24	0.85	1.18	0.71	(0.28–1.85)
							
Other and unspecified malignant neoplasms (XII)	M (9)	17	3	0.50	0.75	0.67	(0.05–9.19)
	F (7)	16	5	4.00	0.57	7.00	(0.57–86.33)
							
Lymphomas (II)	M (110)	205	143	0.96	1.07	0.90	(0.50–1.63)
	F (81)	139	101	1.06	1.24	0.86	(0.41–1.82)
							
Wilms and other renal tumours (VI)	M (27)	47	39	1.05	0.60	1.75	(0.37–8.37)
	F (19)	36	29	0.93	0.40	2.33	(0.39–14.04)
							
Germ-cell, trophoblastic and other gonadal neoplasms (X)	M (85)	148	61	1.10	1.12	0.98	(0.51–1.89)
	F (21)	41	13	1.17	0.75	1.56	(0.41–5.84)

**Figure 1:**
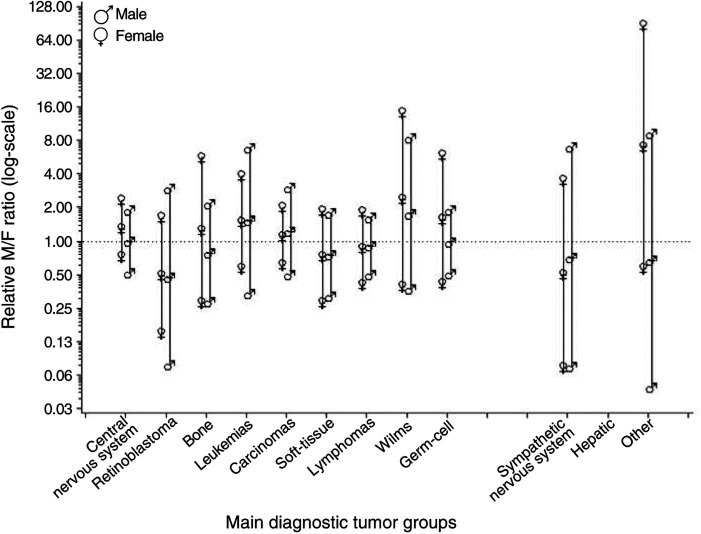
M/F ratio among offspring of irradiated parents relative to the ratio among non-irradiated parents, by the male (♂) and female (♀) survivor sex and main diagnostic tumour group (listed according to increasing estimated gonadal radiation dose; groups with few observations are not included in the ranking but plotted separately). If the postulated genetic effect of radiation occurred, one would have expected the ratios of the M/F ratio among offspring of irradiated to non-irradiated parents to increase for male survivors and decrease for female survivors.

M/F=male-to-female ratio. CI=confidence interval.

a Group number according to the childhood cancer classification (Birch and Marsden, 1987).

presents the M/F ratios among the offspring of childhood cancer survivors, by sex of survivor, main diagnostic tumour group and exposure to radiotherapy. Diagnostic groups are listed according to increasing estimated radiation dose to the gonads. No systematic pattern was observed; that is, no pattern was seen in the M/F ratio among offspring of irradiated parents relative to the M/F ratio among non-irradiated parents in diagnostic tumour groups with presumably high gonadal doses (e.g. lymphomas or Wilms tumour) compared to groups with low gonadal doses (e.g. CNS tumours or retinoblastomas) (Figure 1

). If the postulated genetic effect of radiation occurred, one would had expected the M/F offspring ratio among irradiated parents divided by the M/F offspring ratio among non-irradiated parents to increase for male survivors and decrease for female survivors. There was no significant interaction between radiation and main diagnostic tumour group for either male (*P*=0.99) or female survivors (*P*=0.69). M/F ratios, by survivor sex and estimated gonadal radiation dose, are given in Table 3

**Table 3:** M/F ratios among offspring of childhood cancer survivors, by sex of survivor and estimated gonadal radiation exposure

**Survivor sex (no.)**	**Radiation exposure^a^**	**No. of offspring**	**M/F offspring ratio**	**M/F offspring ratio among irradiatedparents divided by M/F offspring ratioamong non-irradiated parents (95% CI)**	
*Males* (550)	No	665	1.01	1.00 (ref.)^b^	—
	Yes	391	0.96	0.95	(0.74–1.22)
	Low	79	0.88	0.87	(0.55–1.39)
	Low or medium	36	0.80	0.79	(0.40–1.56)
	Medium	67	0.91	0.91	(0.55–1.50)
	Medium or high	144	0.97	0.96	(0.67–1.38)
	High	61	1.10	1.09	(0.65–1.85)
	Unknown	4	3.00	2.97	(0.31–28.7)
					
*Females* (550)	No	720	0.97	1.00 (ref.)^b^	—
	Yes	354	1.07	1.10	(0.85–1.42)
	Low	143	1.07	1.10	(0.77–1.58)
	Low or medium	39	1.05	1.08	(0.57–2.06)
	Medium	26	1.00	1.03	(0.47–2.25)
	Medium or high	101	1.06	1.09	(0.72–1.66)
	High	44	1.10	1.13	(0.61–2.07)
	Unknown	1	—	—	—

M/F=male-to-female ratio. CI=confidence interval.

a Estimate based on information on cancer diagnosis of survivors from the Danish Cancer Registry files with assumptions made with regard to gonadal proximity to likely radiation field.

b Referent category.

. No significant trend according to these categories was observed (male survivors: *P*=0.48; female survivors: *P*=0.51).

Table 4

**Table 4:** M/F ratios among offspring of childhood cancer survivors by sex of survivor, exposure to radiotherapy and interval between cancer diagnosis of parent and birth of offspring

				**M/F offspring ratio among**		
		**No. of offspring**		**M/F offspring ratios**		**irradiated parents divided by M/F offspring ratio**
**Survivor sex (no.)**	**Interval(year)**	**Total**	**Irradiated parents**	**Irradiated parents**	**Non-irradiated parents**	**among non-irradiated parents (95% CI)**	
*Males* (550)	<5	38	10	4.00	1.00	4.00	(0.72–22.3)
	5–9	171	50	0.85	1.09	0.78	(0.41–1.52)
	10–14	250	106	1.04	0.97	1.07	(0.67–1.76)
	15–19	213	91	0.86	0.88	0.98	(0.57–1.68)
	20–24	185	65	1.03	0.76	1.35	(0.74–2.47)
	⩾25	199	69	0.82	1.45	0.56	(0.31–1.01)
							
*Females* (550)	<5	90	21	0.62	0.73	0.85	(0.31–2.31)
	5–9	235	61	1.10	1.07	1.03	(0.57–1.85)
	10–14	255	88	1.20	1.06	1.13	(0.67–1.90)
	15–19	224	76	1.11	0.66	1.68	(0.96–2.93)
	20–24	155	62	1.30	0.82	1.57	(0.82–3.01)
	⩾25	115	46	0.77	2.45	0.31	(0.14–0.69)

M/F=male-to-female ratio. CI=confidence interval.

shows M/F ratios, comparing the ratio of the children of irradiated parents with the children of non-irradiated parents, by interval between cancer diagnosis of the parent and birth of the child. No significant trends were found (male survivors: *P*=0.11; female survivors: *P*=0.18). The only significant finding was a low relative M/F ratio for the offspring of female cancer survivors born 25 or more years after radiation treatment (0.31; 95% CI 0.14–0.69). The ratios of the M/F ratio among offspring of irradiated to non-irradiated male (4.00) and female (0.85) childhood cancer survivors born within 5 years of treatment were in the direction of the postulated genetic effect, but neither was significant.

Information on chemotherapy was judged to be too incomplete in this study to investigate possible effects on the M/F ratio. However, data from a similar patient group included in a Nordic case–control study on second malignant neoplasms after childhood cancer was made available to us (Garwicz *et al*, 2000). Based on a review of the records of 678 childhood cancer patients with various diagnoses, chemotherapy was used to treat all 75 patients diagnosed with leukaemia (I), 78% of patients with Wilms tumour (VI) but only 2% of patients with carcinomas and other malignant epithelial neoplasms (XI) (personal communication with Anderson H). The M/F ratio among childhood cancer survivors in our study with leukaemia (1.41 for male and 1.10 for female survivors) and with Wilms tumour (0.96 for male and 0.80 for female survivors) did not differ from the ratio among survivors with carcinomas (1.27 for male and 0.96 for female survivors) (figures not shown in tables).

## DISCUSSION

Radiation treatment of childhood cancer patients did not alter the sex ratio of their children. No changes in sex ratio were seen for the children of either male or female cancer survivors or over categories of estimated gonadal radiation dose. Experimental evidence (Russell, 1965) suggests that in female mice the yield in mutations, following an exposure experience, may diminish with time either as a consequence of the repair of certain mutations (for a discussion, see Moustacchi, 2000), of cell selection, or both. While the majority of DNA damage is either repaired or fixed as mutations within a few hours or days of exposure, there is evidence that genetic changes may continue to occur for much longer periods of time. It is conceivable, therefore, that the number of sex-linked lethal mutations potentially recoverable could diminish with time and our failure to find a radiation-related effect on the sex ratio reflect this reduction. To examine this thesis, we analysed the changes in the sex ratio over intervals of time between treatment and birth of the children.

The only suggestion of a possible association occurred among children born within 5 years of treatment for both male and female cancer survivors. However, neither of the relative M/F offspring ratios, comparing offspring of irradiated to non-irradiated parents, was close to significant and there was no heterogeneity in the six interval categories except for a reduced relative M/F ratio among children born 25 years or more after maternal treatment. As we would have expected the reduction of the relative M/F ratio among children of irradiated mothers to either diminish with time or at least to stay below 1.0, this significant finding was believed to be a chance finding because of multiple comparisons.

Thus, we find no support for the possibility that high-dose radiation exposures might be associated with an inherited genetic change as indicated by a change in the sex ratio. Reasons for our negative findings might be that the radiation exposure, though large, was not sufficient to result in a detectable genetic effect, that sex ratio is a poor indicator of genetic effects, or that our study had inherent weaknesses that masked a change in the sex ratio.

The typical radiation dose to the gonads following treatment of childhood cancers of patients who later had children were by definition in the non-sterilising range of between 0.01 and 2 Gy, which is similar to the range reported for atomic bomb survivors and much larger than exposures in studies of occupational or environmental circumstances. Our study is consistent with the negative findings in the atomic bomb survivor study (Schull *et al*, 1966) and in two previous studies of survivors of childhood cancer (Hawkins, 1991; Byrne *et al*, 1998). In the most recent and comprehensive study, Byrne *et al* evaluated the sex ratio of 2198 offspring born to male and female survivors treated before 1976, compared with that of 4544 offspring born to controls (1.05 *vs* 1.00, respectively, for males, and 1.01 *vs* 1.05, respectively, for females). Neither difference reached statistical significance. Furthermore, no statistically significant differences were observed comparing survivors treated with potentially mutagenic therapy *vs* those without this treatment.

Other studies of possible genetic effects in humans related to radiation exposure have included groups exposed to ionising radiation received occupationally; for example, nuclear radiation workers and X-ray technologists. These workers receive smaller exposures than the Japanese atomic bomb survivors and childhood cancer survivors. Direct irradiation of the gonads from cancer treatments will result in doses of 10 Gy or more, while scattered radiation for many treatments, such as for the Wilms tumour, could result in testes doses in the order of 0.2–2 Gy. Depending on age, a child treated with cranial radiation receives 0.02–0.2 Gy to the testes (Boice *et al*, 2000; 2002, unpublished data). Other than differences in dose, there are differences in dose rate between workers who are exposed to low dose over many years and atomic bomb survivors and cancer survivors who are exposed to higher does at high dose rates at one point in time or repeated over about 6 weeks. Animal studies show that genetic effects are three times greater when the dose is delivered acutely compared to protracted (Hawkins, 1991; UNSCEAR, 2001). Our study, including patients with high gonadal exposures compared to occupational exposures, does not support previous suggestions of an association of parental exposure with the sex ratio of the offspring.

Our data are also consistent with the latest follow-up of the children of the atomic bomb survivors (Neel and Schull, 1991), which had previously led to a re-interpretation of the value of sex ratio as an indicator of transmitted genetic effects. During the middle of the 20th century, sex determination was thought to be much simpler than is now believed to be the case. X-inactivation and the many sex chromosome abnormalities are known to interfere with gene expression and can influence the proper designation of gender. Such phenomena bring into doubt the validity of the assumption, in humans, that gonadal exposure in males would be expected to increase the M/F ratio and gonadal exposure in females would be expected to decrease the M/F ratio. These simplistic assumptions of radiation effects on parental germ cells is subject to such uncertainty that the sex ratio should not be considered a useful measure of genetic effect. Furthermore, it has been suggested that natural background factors such as conceptions occurring on different days of the menstrual cycle might be associated with marked variations in the sex ratio (Harlap, 1979). Such natural factors might tend to blur observations on sex ratio changes caused by other preconceptional exposures being investigated and further reinforce the statement that sex ratio might not be a particularly good or valid indicator of heritable genetic damage.

Our study has a number of strengths. It is population-based, covering the entire country of Denmark since 1943. Practically all cancer patients alive in 1968 or born thereafter, and their children were identified from accurate population registries. The sex of the child is routinely collected and subject to little uncertainty in reporting. Thus there is little likelihood that selection bias is present that could distort our study results.

Our study, however, also has several limitations. The fact of radiation exposure was taken from notifications in the Danish Cancer Registry for the 1100 survivors included in the study cohort. To validate this assessment, it was compared with information in the medical records on 189 Danish childhood cancer patients included in a previous nested case–control study of second cancers (Garwicz *et al*, 2000). This procedure revealed a relatively high validity of the exposure variable; that is, a high sensitivity (88% of all cases receiving radiotherapy was recorded in the Cancer Registry) and specificity (99% of those receiving no radiotherapy was coded as such in the Registry). Although the registry details seem reliable, misclassification does reduce our ability to detect an association between radiation and sex ratio. We assumed average levels of dose to testes or ovaries based on the type of cancer being treated and the calendar year of treatment. For such cancers as those of brain or kidney, the categorisation is reasonably accurate but for others, such as lymphoma or sarcomas, the range of possible gonadal doses could be broad. Nonetheless, we did not observe any patterns in the offspring sex ratio that suggested changes because of these broad categories of gonadal exposure.

Owing to lack of information, we were unable to examine potential effects on the M/F ratio among offspring of survivors treated with various chemotherapeutic agents or to adjust the effect of radiotherapy on the M/F ratio for these agents. However, no significant dose–response pattern was observed between main diagnostic tumour groups with expected high proportions of patients treated with chemotherapy compared to patients seldom treated with these agents.

Most large-scale studies on parity and parental ages suggest an effect on the sex ratio of birth order and paternal age, and in some studies, also of maternal age (summarised in Jacobsen *et al*, 1999). In a large Danish study of more than 800 000 births, 1980–1993, no independent effect was observed for maternal age or birth order, whereas the sex ratio decreased with paternal age (Jacobsen *et al*, 1999). In our analyses, adjustment for these variables did not affect the results. It has also been argued that the sex determination is not a fully random event. An association with the sexes of previously born children in the family could either be because of *in utero* interaction with the sex of the previous child affecting the sex of the current child (‘Markov association’) or because of a pre-disposition of individuals or couples to have predominantly children of a particular sex (‘Lexis association’) (Jacobsen *et al*, 1999). The large Danish study, however, found no association either with the sex of the preceding child or the combination of sexes of previously born children in the family. Therefore, our statistical analyses were predicated on the supposition that the observations on the sex of offspring within a family are independent.

In conclusion, survivors of childhood and adolescent cancers were not found to have alterations in the sex ratio of their children that could be linked to prior radiation treatments. This may suggest that the human is relatively immune to this type of genetic effect, or, more likely, that alteration in the sex ratio is a poor indicator of genetic effects in humans and should probably not be considered as such, even when gonadal doses are high.
